# A Cycle Ergometer Exercise Program Improves Exercise Capacity and Inspiratory Muscle Function in Hospitalized Patients Awaiting Heart Transplantation: a Pilot Study

**DOI:** 10.5935/1678-9741.20160078

**Published:** 2016

**Authors:** Patrícia Forestieri, Solange Guizilini, Monique Peres, Caroline Bublitz, Douglas W. Bolzan, Isadora S. Rocco, Vinícius B. Santos, Rita Simone L. Moreira, João R. Breda, Dirceu R. de Almeida, Antonio Carlos de C. Carvalho, Ross Arena, Walter J. Gomes

**Affiliations:** 1 Disciplina de Cirurgia Cardiovascular e Cardiologia da Escola Paulista de Medicina da Universidade de São Paulo (EPM-UNIFESP), São Paulo, SP, Brazil.; 2 Departamento de Ciência do Movimento Humana, Escola de Fisioterapia da Universidade Federal de São Paulo (UNIFESP), Santos, SP, Brazil.; 3 Department of Physical Therapy and Integrative Physiology Laboratory, College of Applied Health Sciences, University of Illinois at Chicago, Chicago, USA.

**Keywords:** Heart Failure, Heart Transplantation, Respiratory Mechanics, Muscle Strength/*Physiology, Respiratory Therapy/*Methods

## Abstract

**Objective:**

The purpose of this study was to evaluate the effect of a cycle ergometer exercise program on exercise capacity and inspiratory muscle function in hospitalized patients with heart failure awaiting heart transplantation with intravenous inotropic support.

**Methods:**

Patients awaiting heart transplantation were randomized and allocated prospectively into two groups: 1) Control Group (n=11) - conventional protocol; and 2) Intervention Group (n=7) - stationary cycle ergometer exercise training. Functional capacity was measured by the six-minute walk test and inspiratory muscle strength assessed by manovacuometry before and after the exercise protocols.

**Results:**

Both groups demonstrated an increase in six-minute walk test distance after the experimental procedure compared to baseline; however, only the intervention group had a significant increase (*P*=0.08 and *P*=0.001 for the control and intervention groups, respectively). Intergroup comparison revealed a greater increase in the intervention group compared to the control (*P*<0.001). Regarding the inspiratory muscle strength evaluation, the intragroup analysis demonstrated increased strength after the protocols compared to baseline for both groups; statistical significance was only demonstrated for the intervention group, though (*P*=0.22 and *P*<0.01, respectively). Intergroup comparison showed a significant increase in the intervention group compared to the control (*P*<0.01).

**Conclusion:**

Stationary cycle ergometer exercise training shows positive results on exercise capacity and inspiratory muscle strength in patients with heart failure awaiting cardiac transplantation while on intravenous inotropic support.

**Table:** 

Abbreviations, acronyms & symbols	
**6MWT**	**=Six-minute walk test**
**ATS**	**=American Thoracic Society**
**CR**	**=Cardiac rehabilitation**
**HF**	**=Heart failure**
**MIP**	**=Maximal inspiratory pressure**
**NYHA**	**=New York Heart Association**
**PEB**	**=Perceived exertion Borg**

## INTRODUCTION

Despite significant advances in clinical therapy, heart failure (HF) remains a primary reason for hospitalizations and morbidity and mortality^[1]^. Histological, metabolic and functional adaptations induced by HF promote a decrease in inspiratory and peripheral muscles strength and endurance, leading to exercise intolerance^[1]^. Inspiratory (present in 30% to 50% of the HF patients) and global peripheral muscle weakness^[2]^ are associated with reduced functional capacity, impaired quality of life, and poor prognosis^[3]^.

Consistent scientific evidence has shown that aerobic exercise training is an efficient nonpharmacological tool for HF management^[4,5]^. However, evidence related to exercised-based cardiac rehabilitation (CR) involving hospitalized HF patients requires further examination. In addition, decreased mobility associated with prolonged bed rest during hospitalization could potentiate the detrimental effects of HF^[3]^. For this reason, the early establishment of exercise training is advantageous.

Although optimal drug therapy is initiated in the hospital setting during the acute decompensation period (*i.e.*, intravenous inotropic), many patients become refractory to conventional therapy, requiring consideration for heart transplantation^[6]^. Moreover, after being listed as an appropriate candidate for this surgical procedure, therapeutic management in the pre-transplant phase may be delicate and taxing^[7]^; the main objective during this pre-surgical phase is to avoid further deterioration and death.

Despite the well-established safety and efficacy of exercise training in stable outpatients with HF, the impact of exercise in end-stage HF patients awaiting heart transplantation while on intravenous positive inotropic support remains largely unreported^[8]^. Therefore, the purpose of this study was to evaluate the effects of a stationary cycle ergometer program on exercise capacity and inspiratory muscle function in hospitalized patients awaiting heart transplantation on intravenous inotropic support.

## METHODS

This study was conducted at São Paulo Hospital - Federal University of Sao Paulo. The Institutional Ethics Committee approved this study and a written informed consent was obtained from all patients after explanation of the purpose and procedures of the study.

### Subjects

For this prospective study, patients hospitalized with end-stage HF awaiting heart transplantation were recruited. Patients were considered eligible according to the following criteria: both genders; between 18 and 65 years of age; and with HF diagnosis determined by the referring clinicians on the basis of clinical presentation [New York Heart Association (NYHA) classes III and IV] and confirmed by echocardiography.

Patients with unstable angina pectoris, acute coronary syndromes, atrial and ventricular arrhythmias leading to hemodynamic compromise, chronic renal failure or dialysis, neuromuscular and psychiatric conditions that could potentially interfere with performance on the 6-minute walk test (6MWT), and those in need of non-invasive ventilation support during exercise were excluded. Patients with chronic lung disease confirmed by pulmonary function testing according to the American Thoracic Society (ATS) were also excluded^[9]^.

All patients received an individually optimized medical treatment, including intravenous inotropic support, angiotensin-converting enzyme inhibitors, aldosterone antagonists and other diuretics.

### Randomization and Blinding Procedures

The randomization procedure was performed through a computer program and allocation secrecy was kept by numbered, sealed, opaque envelopes. The patients were randomized into two groups: 1) Control Group (n=11) - conventional protocol; and 2) Intervention Group (n=7) - stationary cycle ergometer exercise program.

### Procedures

The study protocol was initiated 24 hours after hospital admission or clinical stabilization, and consisted of two different exercise protocols, applied twice a day during hospitalization. Regardless of group allocation, all patients were able to walk unaided for short distances (*e.g.*, from the bed to the bathroom) and were instructed to sit upright in a chair at least twice a day outside of sessions associated with the protocol.

Patients were familiarized with a perceived exertion Borg (PEB) scale prior to each exercise session, and were instructed to exercise at a PEB scale rating of 3-4 ('moderate' to 'somewhat strong').

### Study Protocols

Control Group - each session consisted of breathing exercises and global active exercises of the upper and lower limbs while in the upright seated position.

**Fig. 1:**
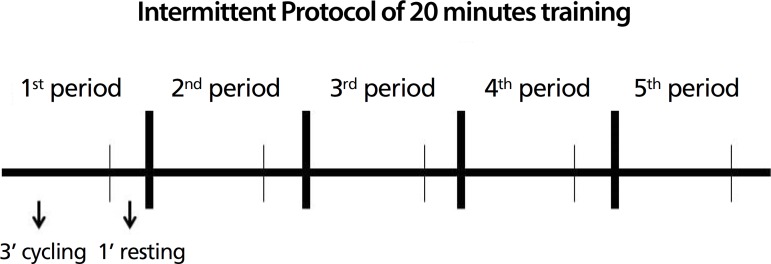
Intermittent protocol: stationary cycle ergometer exercise training.

Intervention Group - each session included stationary cycle ergometer exercise (Mini bike E5, Acte Brazil - lower extremity) while in the upright seated position for 20 minutes. The protocol was performed intermittently with 5 periods; each period consisted of 3 minutes of cycling followed by 1-minute of rest (Figure 1).

### Measurements and Outcomes

#### Inspiratory Muscle Strength Evaluation

Evaluation of respiratory muscle strength consisted of measuring the maximal inspiratory pressure (MIP) by an analog manovacuometer (GerAr med^®^. The recordings were performed for both pre and post training protocols. Each maneuver was performed five times with 1 minute rest between them, and sustained pressure during 2 seconds by the patient was recorded according to established guidelines^[10]^. MIP was measured from residual volume; the patient was requested to perform a forced expiration and then take a maximal inspiratory effort against an occluded airway (Mueller maneuver). The prediction equation proposed by Neder et al.^[11]^ was used to predict maximal inspiratory pressure for all patients.

#### Submaximal Functional Capacity Assessment

All patients underwent a 6MWT to assess their baseline submaximal functional capacity, which was performed according to ATS standards^[12]^ by an evaluator blinded to group allocation. The prediction equation proposed by Iwama et al.^[13]^ was used to predict walking distances for all patients. The follow-up 6MWT was conducted approximately 3 weeks later, immediately following protocol completion. During the test, all patients were on intravenous inotropic support administered by an infusion pump, which was carried out by the same blinded evaluator who walked at the patient's side.

#### Exercise Interruption Criteria

The exercise protocol or the 6MWT was discontinued if the subject presented signs of exercise intolerance, such as low cardiac output (*i.e.*, cyanosis, pallor and nausea), bradycardia, a drop in systolic blood pressure of >15 mmHg in comparison to baseline, an excessive rise in systolic blood pressure defined as > 200 mmHg, a rise in diastolic blood pressure during exercise >110 mmHg, chest pain or symptoms of fatigue.

#### Statistical Analysis

Data were reported as mean ± standard deviation. Normal distribution for all variables was verified using the Kolmogorov-Smirnov test. When variables were compared between groups, the unpaired Student's t-test and Mann-Whitney test were used as appropriate. For intragroup analysis, the paired Student's t-test was used. Categorical variables were analyzed by the Pearson Chi-square test. The Pearson correlation coefficient was used to evaluate associations of interest. A *P*-value of <0.05 was considered statistically significant for all tests. Statistical analyses were performed using GraphPad Prism 6.0 Software (GraphPad Software Inc, San Diego, CA, USA).

## RESULTS

**Fig. 2:**
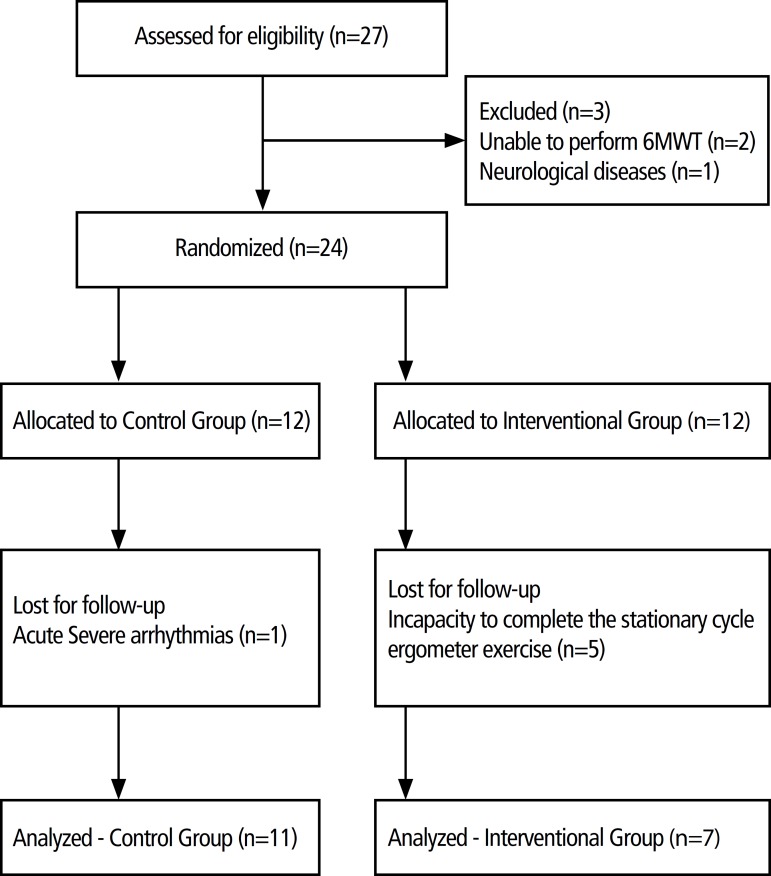
Flowchart of subject recruitment, enrollment, randomization and completion. 6MWT= 6-minute walk test.

A flow-chart illustrating progression of patients through the study is shown in Figure 2. The groups were homogeneous and no statistical difference was found when comparing baseline demographic and clinical characteristics, as shown in Table 1.

**Table 1:** Patients’ baseline characteristics.

Variables	Control Group (n=11)	Intervention Group (n=7)	*P* value
Age (years)	45±11.2	48.3±10.2	0.44
Female/Male(n)	02/9	2/5	0.60
BMI (kg/m^2^)	22.3±3.0	24.2±3.1	0.08
Etiology (n)	7 ischemic	5 ischemic	0.35
	1 idiopathic	1 idiopathic	
	3 hypertensive	1 non-compact myocardium	
ICD / pacemaker (n)	3	2	0.55
LVEF	0.36±0.03	0.33±0.03	0.22
**Pulmonary function**			
FVC (L)	3.2±8.6	3.5±7.8	0.65
% predicted	83.9±15.08	85.4±16.8	
FEV_1_(L)	2.78±7.2	2.5±6.3	0.34
% predicted	88.5±11.7	86±13.9	
MIP (cmH_2_O)	57.5±10.2	60±15.1	
% predicted	52.6±10.6	54.4±18.2	
**Drug Therapy**			
ACE inhibitors (mg/d)	22.3±11.2	23.20±9.3	0.37
Furosemide (mg/d)	28.7±8.9	27±9.2	0.22
Dobutamine (mcg/kg/min)	9.18±5.84	8.78±6.8	0.55
Beta-blocker (mg/d)	28.12±10	29.14±11	0.42
**Laboratory tests**			
NT-Pro-BNP(pg/ml)	11.789±2.576	10.897±1.998	0.44
**Functional Capacity**			
6MWT (meters)	321.2±44	310±51.2	0.55
% predicted	55.2±10	61±9.57	
**Length of exercise sessions (days)**	19.4±3.5	22.3±4.5	0.19

Values expressed as mean ± standard deviation ACE=angiotensin converting enzyme; BMI=body mass index; ICD=implanted cardioverter defibrillator; FEV1=forced expiratory volume in 1 second; FVC=forced vital capacity; LVEF=left ventricular ejection fraction; MIP =maximal inspiratory pressure; NT-pro- BNP=Amino-terminal pro-brain natriuretic peptide; 6MWT=6-minute walk test

In the overall cohort, before initiation of the study protocol, a significant positive correlation was observed between 6MWT distance and inspiratory muscle strength (r=0.58; *P*<0.02).

The assessment of functional capacity, for both groups, demonstrated an increase in 6MWT distance after the experimental procedure compared to the baseline. However, only the intervention group had a statistically significant increase (*P*=0.08 and *P*=0.001 for the control and intervention groups, respectively). Intergroup comparison showed an increase of 15.5% (48 meters) in the intervention group compared to control (16 meters) after completion of the study protocol (*P*<0.001) (Table 2).

**Table 2:** Functional capacity and maximal inspiratory pressure between groups after protocol.

Variables	Control Group (n=11)	Intervention Group (n=7)	*P*
6MWT (m)	337.2±37.2	358±42.4	<0.001
% pre	104.9±2	115.5±32	
MIP (cmH_2_O)	57.5±12	69.6±14.1	< 0.01
% pre	107±22.2	115±24.2	

Values expressed as mean ± standard deviation. *P*value refers to the difference between the groups. % pre=considering 100% of the baseline value pre-protocol; 6MWT=6-minute walk test; MIP=maximal inspiratory pressure

In regard to the inspiratory muscle strength evaluation, the intragroup analysis demonstrated an increased strength after the protocols compared to baseline for both groups. However, a statistically significant difference was only demonstrated in the intervention group (*P*=0.22 and *P*<0.01, respectively). Intergroup comparison revealed a significant increase of 15% in the intervention group compared to the control group after the study protocol (*P*<0.01) (Table 2).

## DISCUSSION

The findings of the current study indicated that a stationary cycle ergometer exercise training program positively and significantly impacted functional performance and respiratory muscle strength in end-stage HF patients on continuous inotropic support awaiting heart transplantation.

Few data are available regarding the effects of exercise training in hospitalized patients with advanced HF. In addition, there are gaps in the literature regarding the impact of exercised-based CR in pre-cardiac transplantation patients while receiving continuous inotropic infusion. To our knowledge, this is the first pilot study demonstrating the effects of a stationary cycle ergometer exercise training program on functional capacity and inspiratory muscle strength in HF patients with this end-stage condition.

Previous studies showed that inspiratory muscle weakness is closely linked to the deterioration of cardiac function, the impairment of the ability to exercise and disease severity (NYHA class)^[14]^.

Several mechanisms are responsible for respiratory muscle weakness in patients with HF, among them a reduction in the total number of cross-bridges between actin and myosin and muscle cross-sectional area in the diaphragm^[15,16]^. Skeletal muscle dysfunction induced by HF pathophysiology and compounded by physical inactivity can result in a substantial decrease in strength and endurance of the respiratory musculature. Meyer et al.^[17]^ showed that patients with a MIP greater than -70 cmH_2_O had a higher one-year mortality rate when compared to patients with a MIP lower than -90 cm H_2_O. Thus, dysfunction of the respiratory musculature is considered an independent predictor of poor prognosis in patients with HF^[17]^. In the current study, when the data were analyzed in the overall cohort at baseline, the patients showed a MIP greater than -70 cmH_2_O and a significant positive correlation was observed between 6MWT distance and inspiratory muscle strength. Therefore, patients with a low 6MWT distance had a higher likelihood of poor inspiratory muscle strength.

Evidence indicates that some performance modification on the 6MWT following an intervention reflects changes in clinical functional status^[18]^. In this study, to evaluate functional capacity, all patients underwent a 6MWT. This test is a simple, practical and safe form of evaluation in HF patients, particularly those who are hospitalized, and demonstrate advanced disease severity. In regard to submaximal functional capacity, our results demonstrate that both groups obtained an increase in 6MWT distance after the protocol compared to baseline; however, only the intervention group had a statistically significant increase. When comparing groups, after the study protocol, those participating in the cycle ergometer exercise program showed a statistically significant greater increase (48 meters) in 6MWT distance compared to the control group in relation to baseline. Previous studies have reported that 6MWT distance indicates changes in a patient's functional status. A recent study found that a change of 45 meters during the 6MWT indicates a minimal clinically significant improvement following rehabilitation in patients with HF^[19,20]^. Thus, in the present study, only the cycle ergometer exercise program group reached a meaningful clinical improvement in 6MWT distance.

A positive impact on functional capacity and quality of life in outpatients with HF following exercise training has been clearly established^[21-24]^. For the in-hospital phase, limited published literature shows that early mobilization with daily cycle ergometer exercise training was able to improve quadriceps strength and functional capacity in critically ill inpatients subject to prolonged bed rest^[25]^. For HF inpatients, a case report used a progressive, semi-independent interval-walking program in a patient with advanced HF on continuous dobutamine therapy showed functional improvements and the clinical utility of the 6MWT^[26]^. Similar results were reported in another case report that used ambulation sessions and seated lower extremity ergometer sessions^[27]^. Moreover, Arena et al.^[8]^ demonstrated in a case report that exercise training consisting of ambulation on a treadmill, lower extremity ergometer, and resistive exercises improved aerobic exercise time and sustainable exercise workload in an inpatient awaiting heart transplantation while on intravenous positive inotropic support. Our study adds to this limited body of literature, as it was the first pilot study to demonstrate that an exercised-based training program could bring about positive and statistically significant improvements in end-stage HF patients awaiting cardiac transplantation.

Changes in the inspiratory musculature play an important role in the pathophysiologic milieu induced by HF and lead to oftentimes substantial exercise limitations^[17]^. There is a compelling body of evidence that indicates the degree of respiratory muscle weakness is related to the severity of HF, functional capacity limitations, and diminished quality of life^[15-17,28,29]^. It is important to highlight that exercise training leads to a notable improvement in ventilatory capacity, independent of the type of program - endurance exercise performed either with constant load intensity or with interval training, combining different periods of intensity or a program that includes resistance training sessions^[30]^. There are studies showing that aerobic training alone can prevent diaphragm tumor necrosis factor-α (TNF-α)-induced loss of force and significantly improve MIP in patients with HF^[31]^. Ours results are consistent with other studies and revealed that the intervention group had a statistically significant increase between baseline and follow-up MIP evaluation, reflecting improved inspiratory muscle strength.

Therefore, this study demonstrated that a stationary cycle ergometer exercise training program could improve functional capacity and inspiratory muscle function in hospitalized patients awaiting heart transplantation while on inotropic support. These results support a new standard approach to the management of this HF condition, benefiting the patient in terms of quality of life, functional capacity, and post-surgical trajectory.

We speculate that the observed improvement in functional capacity and inspiratory muscles function could aggregate to attain better operative outcomes at the time of the heart transplantation surgery.

There are some important limitations to this study. Our investigation included a low number of patients, despite being the largest so far. In the intervention group, 5 patients were excluded due to incapacity to complete the stationary cycle ergometer exercise program without non-invasive ventilation support. Therefore, future research is needed to further assess this type of exercise programs in HF patients with these clinical characteristics to further support the findings reported herein.

## CONCLUSION

This current study demonstrates that a stationary cycle ergometer exercise program leads to positive improvements in functional capacity and inspiratory muscle strength in patients with HF who are hospitalized and awaiting heart transplantation while on intravenous inotropic support.

**Table:** 

Authors’ roles & responsibilities	
PF	Conception and design study; manuscript redaction or critical review of its content; final manuscript approval
SG	Conception and design study; manuscript redaction or critical review of its content; final manuscript approval
MP	Manuscript redaction or critical review of its content; final manuscript approval
CB	Manuscript redaction or critical review of its content; final manuscript approval
DWB	Manuscript redaction or critical review of its content; final manuscript approval
ISR	Manuscript redaction or critical review of its content; final manuscript approval
VBS	Manuscript redaction or critical review of its content; final manuscript approval
RSLM	Manuscript redaction or critical review of its content; final manuscript approval
JRB	Manuscript redaction or critical review of its content; final manuscript approval
DRA	Manuscript redaction or critical review of its content; final manuscript approval
ACCC	Manuscript redaction or critical review of its content; final manuscript approval
RA	Manuscript redaction or critical review of its content; final manuscript approval
WJG	Manuscript redaction or critical review of its content; final manuscript approval
